# Supplementary material for: In Vitro Systematic Drug Testing Reveals Carboplatin, Paclitaxel, and Alpelisib as a Potential Novel Combination Treatment for Adult Granulosa Cell Tumors
Source: Cancers (Basel). 2021 Jan 20;13(3):368. doi: 10.3390/cancers13030368 (PMC7864192; doi:10.3390/cancers13030368)
Supplement: Supplementary file 1 [file cancers-13-00368-s001.zip › cancers-1027226-supplementary (1).pdf]

Figure S1. Copy number profiles of *FOXL2* wildtype and hemizygous mutant cell lines

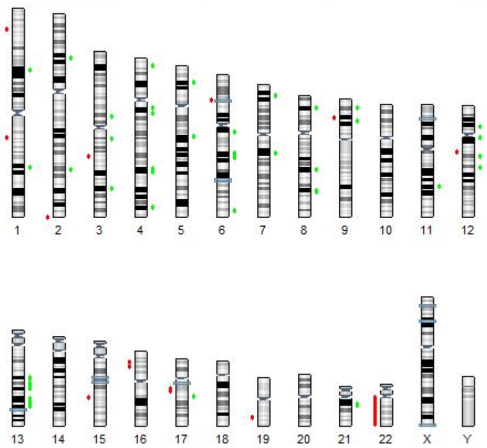

GCPA096.Tumor.T1.II
FOXL2 +/-

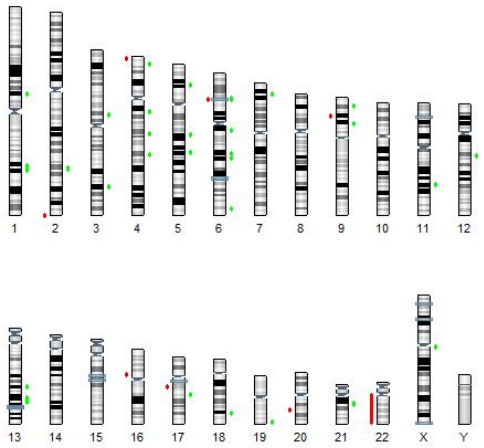

GCPA096.Cell.T1.II
FOXL2 -/-

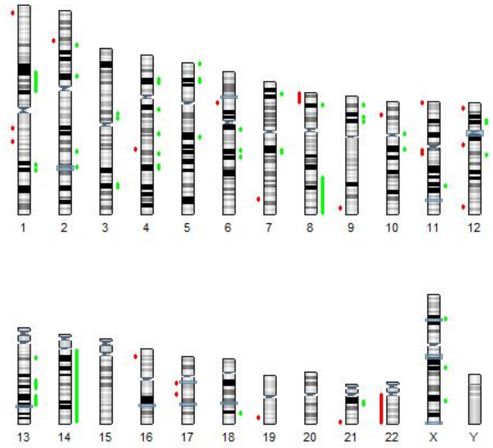

GCPA113.Tumor.T1.I
FOXL2 +/-

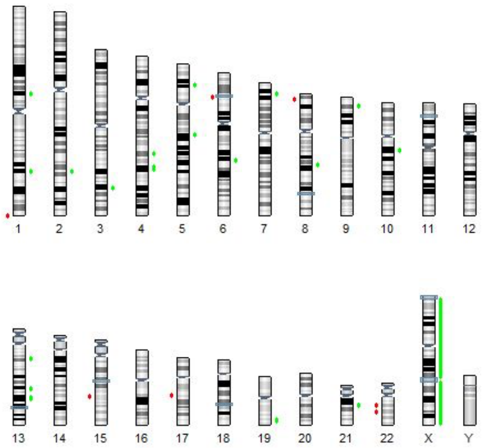

GCPA113.Cell.T1.I
FOXL2 -/-

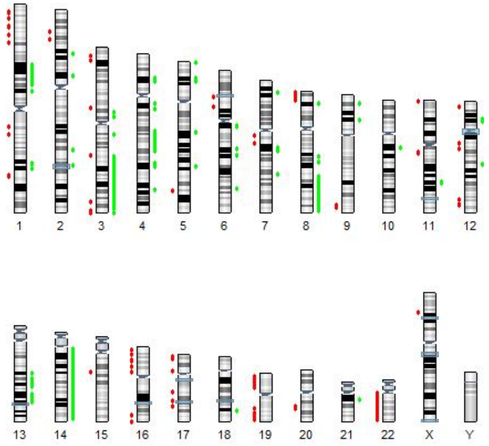

GCPA113.Tumor.T1.II
FOXL2 +/-

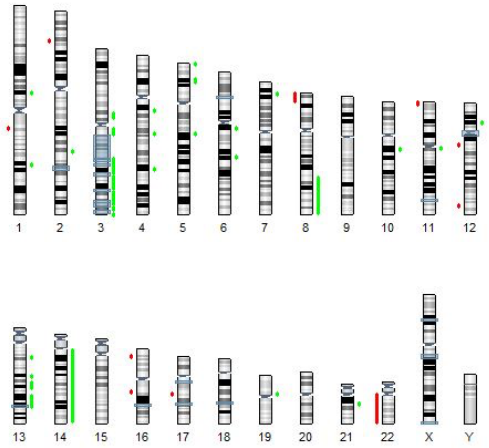

GCPA113.Cell.T1.II
FOXL2 +/+

Figure S2. Drug screen results of control cell lines

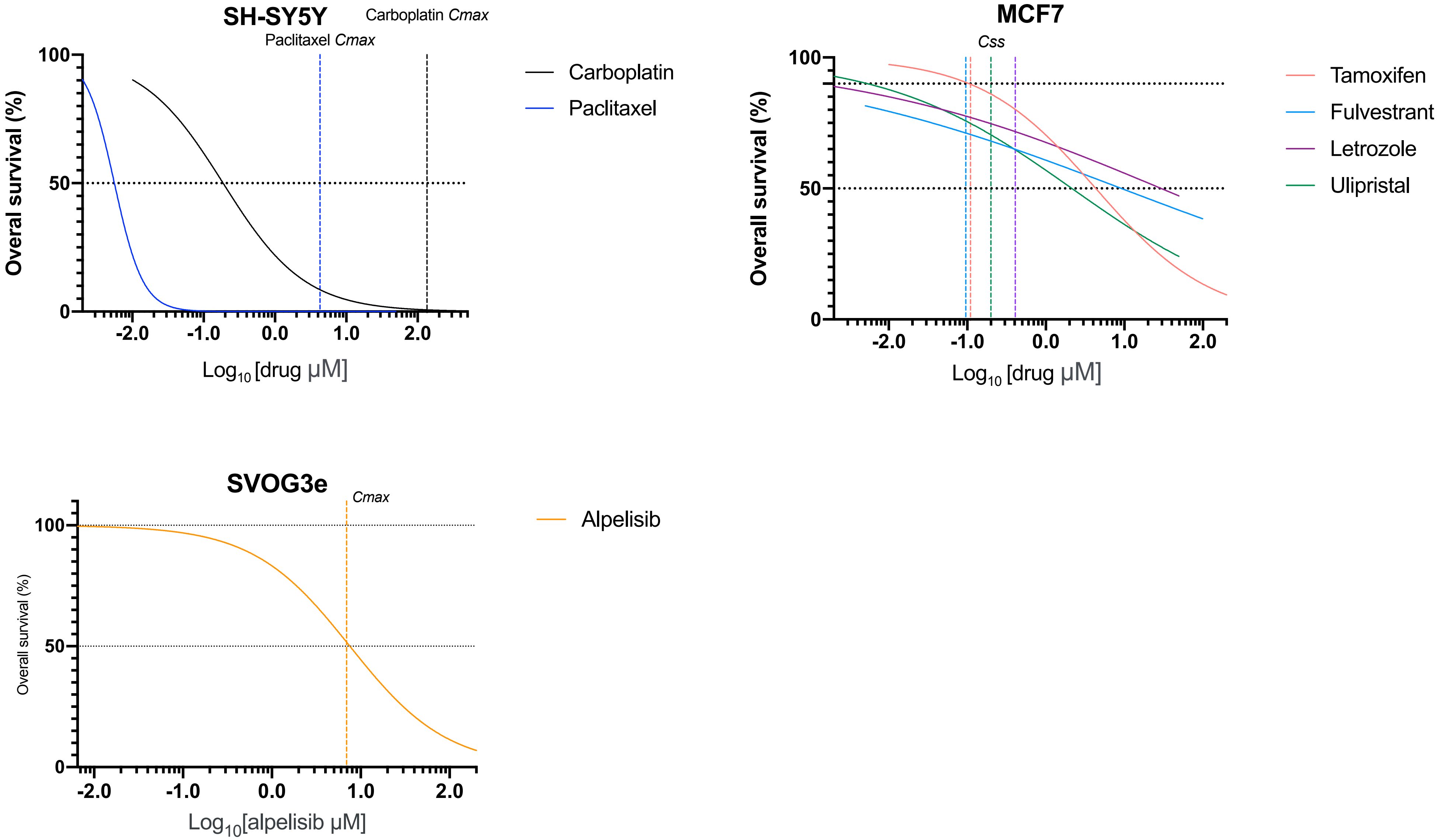

Figure S3. Monotherapy drug screen results

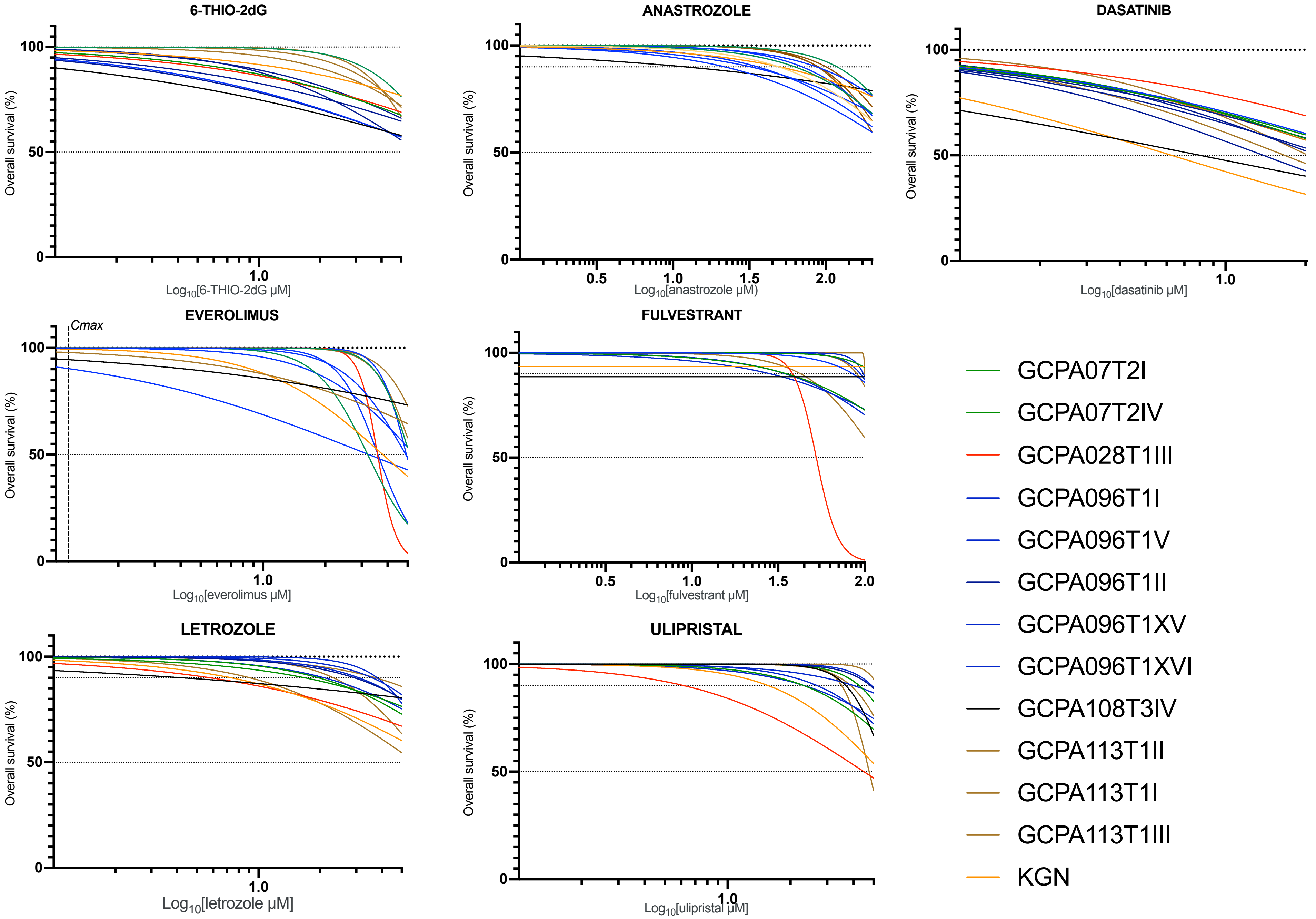

Figure S4. R-squared values for monotherapy response curves

	KGN	GCPA007T2I	GCPA007T2IV	GCPA028T1II	GCPA096T1I	GCPA096T1II	GCPA096T1V	GCPA096T1XV	GCPA096T1XVI	GCPA113T1II	GCPA113T1I	GCPA113T1III	GCPA108T3IV
6THIO2dG	0,6177	0,9213	0,9863	0,9386	0,957	0,965	0,9787	0,9507	0,9787	0,9817	0,9796	0,9908	0,7288
Alpelisib	0,9657	0,9674	0,9879	0,971	0,9948	0,9841	0,9908	0,9945	0,9767	0,9324	0,9669	0,9798	0,9934
Anastrozole	0,7697	0,9609	0,994	0,93	0,9805	0,9891	0,9761	0,9866	0,9297	0,9369	0,9747	0,8847	0,5915
Carboplatin	0,8334	0,9485	0,9832	0,9908	0,9943	0,981	0,9413	0,9286	0,892	0,964	0,9513	0,984	0,8898
Dasatinib	0,797	0,9272	0,9298	0,9195	0,9422	0,9288	0,9335	0,9341	0,9639	0,8976	0,9346	0,9478	0,5136
Everolimus	0,8376	0,943	0,9417	0,9715	0,8015	0,9033	0,8996	0,9537	0,8998	0,9586	0,9837	0,9495	0,5885
Fulvestrant	Interrupted	0,9931	0,9446	0,9691	0,9608	0,9921	0,9822	0,9808	0,9899	Interrupted	0,9793	0,9899	Interrupted
Letrozole	0,7844	0,9661	0,9673	0,955	0,9784	0,9847	0,9732	0,9711	0,9905	0,8128	0,9639	0,9845	0,2773
Paclitaxel	0,6931	0,9732	0,9384	0,9706	0,986	0,9736	0,936	0,9743	0,9767	0,9592	0,9747	0,5125	0,3109
Tamoxifen	0,9327	0,9915	0,9795	0,9276	0,9894	0,9943	0,984	0,9669	0,9932	0,9772	0,9524	0,9653	0,8594
Ulipristal	0,8916	0,9957	0,974	0,967	0,9175	0,9802	0,9564	0,9768	0,9492	0,9976	0,9569	0,9473	0,6503

Mean	0,90
Median	0,96
Max	1,00
Min	0,13

Figure S5. R-squared values for combination treatment response curves

Combination Ratio	Car+Pac (10:1)	Car+Pac+Let (10:1:1)	Car+Pac+Tam (10:1:0.4)	Car+Pac+Uli (10:1:1)	Car+Pac+Ful (10:1:2)	Car+Pac+Ana (10:1:4)	Car+Pac+Eve (10:1:1)	Car+Pac+Alp (10:1:2)	Car+Pac+THIO (10:1:1)	Car+Pac+Das (10:1:0.4)	Eve+Alp (1:2)	Eve+Tam (5:2)
KGN	0,8258	0,7584	0,797	0,4999	0,4949	0,5415	0,4589	0,8771	0,5531	0,8661	0,6349	0,8518
GCPA007T2I	0,8586	0,7579	0,8376	0,9021	0,7365	0,6262	0,828	0,9198	0,4953	0,6114	0,9484	0,9128
GCPA007T2IV	0,9057	0,3885	0,8986	0,8742	0,4175	0,819	0,8433	0,9581	0,5895	0,8296	0,8709	0,9032
GCPA028T1II	0,8361	0,9309	0,8208	0,3823	0,9209	0,6411	0,821	0,9174	0,2462	0,7453	0,865	0,9534
GCPA096T1I	0,8618	0,8687	0,7753	0,6291	0,7088	0,9583	0,7279	0,9427	0,887	0,8984	0,9572	0,8806
GCPA096T1II	0,9554	0,8386	0,8388	0,5153	0,559	0,8642	0,8187	0,9822	0,8921	0,8699	0,993	0,6205
GCPA096T1V	0,7069	0,4959	0,8759	0,4689	0,6153	0,8923	0,6956	0,9876	0,8905	0,7637	0,9557	0,8263
GCPA096T1XV	0,7926	0,6781	0,7275	0,7261	0,6486	0,7436	0,7094	0,9642	0,8436	0,8362	0,9113	0,849
GCPA096T1XVI	0,7245	0,6535	0,8435	0,5424	0,8224	0,4463	0,7931	0,8603	0,6152	0,5518	0,8111	0,9663
GCPA113T1I	0,9421	0,9054	0,8225	0,9044	0,9368	0,8995	0,8089	0,9	0,7275	0,6533	0,918	0,8197
GCPA113T1II	0,768	0,8083	0,9235	0,9085	0,8066	0,829	0,932	0,9592	0,8244	0,9105	0,8783	0,6328
GCPA113T1III	0,6723	0,4819	0,758	0,9248	0,8548	0,8356	0,8053	0,9551	0,7658	0,7303	0,9331	0,8447
GCPA108T3IV	0,2679	0,4068	0,7524	0,5066	0,5935	0,436	0,4127	0,6085	0,4691	0,6788	0,9731	0,3021

Mean	0,76
Median	0,82
Max	0,99
Min	0,25
